# Expression of Heat Shock Protein (Hsp90) Paralogues Is Regulated by Amino Acids in Skeletal Muscle of Atlantic Salmon

**DOI:** 10.1371/journal.pone.0074295

**Published:** 2013-09-06

**Authors:** Daniel Garcia de la serrana, Ian A. Johnston

**Affiliations:** Scottish Oceans Institute, School of Biology, University of St Andrews, Fife, Scotland, United Kingdom; University of Geneva, Switzerland

## Abstract

Heat shock proteins 90 (Hsp90) have an essential role in sarcomere formation and differentiation in skeletal muscle and also act as molecular chaperones during protein folding impacting a wide range of physiological processes. We characterised and provided a phylogenetically consistent nomenclature for the complete repertoire of six Hsp90 paralogues present in duplicated salmonid fish genomes (Hsp90α1a, Hsp90α1b, Hsp90α2a, Hsp90α2b, Hsp90ß1a and Hsp90ß1b). The expression of paralogues in fast skeletal muscle was investigated using *in vivo* fasting-feeding experiments and primary myogenic cultures. Fasted juvenile Atlantic salmon (*Salmo salar*) showed a transient 2 to 8-fold increase in the expression of all 4 Hsp90α paralogues within 24h of satiation feeding. H*sp90α1a* and *hsp90α1b* also showed a pronounced secondary increase in expression after 10 days, concomitant with muscle differentiation and the expression of *myogenin* and sarcomeric proteins (*mlc2*, *myhc*). Hsp90ß1b was constitutively expressed whereas *Hsp90ß1a* expression was downregulated 10-fold between fasted and fed individuals. Hsp90α1a and Hsp90α1b were upregulated 10 to 15-fold concomitant with myotube formation and muscle differentiation *in vitro* whereas other Hsp90 paralogues showed no change in expression. In cells starved of amino acid (AA) and serum for 72h the addition of AA, but not insulin-like growth factor 1, increased phosphorylation of mTor and expression of all 4 hsp90α** paralogues and associated co-chaperones including *hsp30*, *tbcb*, *pdia4*, *pdia6*, *stga* and *fk504bp1*, indicating a general activation of the protein folding response. In contrast, Hsp90ß1a expression *in vitro* was unresponsive to AA treatment indicating that some other as yet uncharacterised signal(s) regulate its expression in response to altered nutritional state.

## Introduction

The cytosolic Hsp90s (Hsp90AA1 and Hsp90AB1) are some of the most abundant proteins in many cell types, representing around 2% of the soluble proteins in non-stressed cells. Hsp90α (Hsp90AA1) and Hsp90ß (Hsp90AB1) are considered to have originated by duplication in the vertebrate common ancestor around 500 million years (MY) ago [1,2]. Almost 200 different proteins are thought to require Hsp90s to achieve their final configuration including kinases [3], growth factor receptors [4], transcription factors [5] and a remarkable number of oncogenic proteins [6]. Hsp90s are therefore key regulators in cell physiology and are involved in diverse processes including signal transduction, protein folding, morphology and differentiation. Hsp90s function and client specificity is regulated by interaction with a group of non-client binding partners, known as co-chaperones, that includes Cdc37, p23, Fk506bp1, Chip, Sgta, Aha1 or Unc45 [7,8].

Three main functional domains have been described for Hsp90: the N-terminal domain, the flexible middle region and the C-terminal domain [9,10]. The N-terminal region contains the ATP binding domain with ATPase activity. The hydrolysis of ATP initiates a cycle that modifies Hsp90 configuration and acts as a molecular motor for the folding process (for a complete description see 9). Hsp90 chaperone function as a homodimer, with the C-terminal domain and middle region being responsible for dimerization [9,10].

Several studies have highlighted the importance of Hsp90 for skeletal muscle embryogenesis and myofibrillogenesis involving direct interaction with the myoblast determination factor MyoD [3,11]. Inhibition of Hsp90 function in C2C12 cells induced a significant decrease in myogenin and Akt expression, and caused disruption of the MyoD-Cdc37-Hsp90 complex, inhibiting myotube formation [12]. It is also known that Hsp90 regulates myosin heavy chain motor domain folding, one of the main components of the sarcomere, by interaction with the co-chaperone Unc45 [13,14].

Teleosts have undergone whole genome duplication (WGD) relative to the ancestor of tetrapods (Jaillon et al. 2004). The zebrafish (*Danio rerio*) genome contains *Hsp90α1* and *Hsp90α2* genes. Functional studies demonstrated that inhibition of *hsp90α1* expression strongly perturbed embryonic muscle development, whilst blocking *hsp90*α*2* had no effect, suggesting sub-functionalization between paralogues [15]. Several studies have described increased expression of *hsp90* in response to temperature stress, although the issue of gene paralogues is generally ignored [16–18]. More recently, a dramatic and rapid increase in *hsp90α* expression has been reported in fish fed to satiation following a period of dietary restriction though the signals involved have not been investigated [19–22].

Atlantic salmon and other salmonid fish underwent a second lineage-specific whole genome duplication event around 88 million years ago followed by gene loss [23,24]. Potentially two copies of each zebrafish gene may be present in the Atlantic salmon genome [23]. The aim of the present study was to characterise the complete repertoire of Hsp90α and Hsp90β paralogues in salmonid genomes and determine their expression in a fasting-refeeding model using juvenile Atlantic salmon (*Salmo salar* L.). To provide further insight into the transcriptional regulation of this gene family we used primary myogenic cell cultures and investigated the effects of amino acid and IGF1 treatments on Akt and mTor phosphorylation and the expression of Hsp90 paralogues and co-chaperones.

## Methods

### Ethics statement

This study was conducted on Atlantic salmon (*Salmo salar* L.) of aquaculture origin. Fish were humanely killed by a blow to the head following Schedule 1 of the Animals (Scientific Procedures) Act 1986 (Home Office Code of Practice. HMSO: London January 1997), and all husbandry procedures and experimental protocols were approved by University of St Andrews Animal Ethics and Welfare Committee.

### Hsp90 sequencing

Previously sequenced Atlantic salmon Hsp90α and Hsp90β (NM_001173702 and NM_01146473 respectively) were blasted against the Atlantic salmon genome draft deposited in the NCBI genome database (ASM23337v1). Four sequences similar to Hsp90α and two to Hsp90β were found and read trace quality was checked using the Trace Archive Nucleotide BLAST from NCBI. All sequences were experimentally confirmed as follows: primers against the start and stop regions (Table S1) were used to amplify Hsp90 paralogues from a cDNA mix of different Atlantic salmon tissues (fast muscle, liver, slow muscle, brain, skin, gills, heart, liver, fat, kidney and myoblast). Specific bands in the right size range (around 2.2kb) were cloned as a whole in TOPO^®^ plasmid (Invitrogen), transformed by thermal shock into *E. Coli* Top10 (Invitrogen) and grown in LB-Agar plates with 75ng/ml ampicillin. 10-12 clones per product were sent for SANGER sequencing to the Dundee Sequencing Services. Hsp90 paralogues sequences were submitted to the GenBank database: KC150878, KC150879, KC150880, KC150881, KC150882 and KC150883 (Table S1). Hsp90 paralogues sequences were conceptually translated to amino acid using Virtual ribosome [25] and aligned with MAFFT version 6 [26] (Figure S1).

### Rainbow trout transcriptome

Ten Rainbow trout specific 454-Titanium libraries from different sources were retrieved from the Sequence Reads Archive database (SRA): SRX041526-31 (skeletal fast muscle), SRX085156 (stressed Rainbow trout), DRX000493 (adipose tissue), SRX007396 (non-stressed Rainbow trout) and SRX041532-7 (larvae trunks). A total of 6,154,973 reads were *de novo* assembled by Newbler 2.5 assembly software (Roche, 454 Life Sciences). Assemblies were compiled using a Debain Linux system, IBM x3755 8877, with 8 CPU cores (4 x dual-core AMD Opteron), 64-bit, 2.8 GHz processor with 128 Gb of RAM maintained by the University of St Andrews. Hsp90 sequences were retrieved from the rainbow trout transcriptome by comparison with the Atlantic salmon paralogues using BioEdit free software [27].

### Phylogenetic and synteny analysis

Teleost Hsp90α (Hsp90AA1) and Hsp90β (Hsp90AB1) sequences were retrieved for *Oryzias latipes*, *Danio rerio*, *Oreochromis* *niloticus* , *Gadus* *moruha* , *Gasterosteus* *aculeatus* , *Takifugu* *rubripes* and *Tetraodon nigroviridis* from the Ensembl database [28]. A total of 37 coding sequences, including Atlantic salmon and rainbow trout, were aligned using GUIDENCE webserver [29]. Only aligned regions with a GUIDENCE quality score over 0.93 were used for further analysis. Maximum Likelihood (ML) and Neighbour-Joining (NJ) trees with 500 bootstraps replications were constructed using MEGA5 [30] with Jone-Taylor-Thorton (JTT) and Gamma distribution as the evolutionary model. Trees generated were visualized and edited using Fig tree v 1.3.1.

Synteny surrounding Hsp90 genes was manually inferred by study of Ensembl genome assemblies of *Danio rerio,* *Gasterosteus* *aculeatus* *, Oryzias latipes,* *Oreochromis* *niloticus* *,* *Tetraodont* *nigroviridis* *,* *Takifugu* *rubripes* *, Gadus morhua,* *Xiphophorus* *maculatus* *, Homo sapiens,* *Xenopus* *tropicalis* and *Meleagris* *gallopavo* [28].

### Atlantic salmon fasting refeeding experiment

A total of 150 Atlantic salmon juveniles (59.8 ± 7.9 g; mean ± SD) were obtained from Landcatch Natural Selection Ltd and maintained at ~10.6^o^C in replicate tanks at the Scottish Oceans Institute. Fish were fed a maintenance diet for 21 days and then fasted for 7 days and fed to satiation with commercial feed (EWOS) supplemented by bloodworms. Fast skeletal muscle was dissected from dorsal epaxial at 0.6 standard length at day 0 (after 7 days of fasting) and 1, 3, 5, 8, 15 and 21 days of satiation feeding.

### Atlantic salmon primary myogenic cell culture

Myoblast cell culture was performed as previously described [31]. In brief, myogenic cells were extracted from fast skeletal muscle and maintained in 10% (v/v) Foetal Bovine Serum (SIGMA) DMEM (SIGMA) medium containing antibiotics (SIGMA) for 16 days. Cells RNA was extracted after 2, 4, 6, 8, 10, 12, 14 and 16 days after plating using RNeasy mini kit (QIAGEN). For immunofluorescence, cells were fixed in 4% (m/v) paraformaldehyde in PBS (SIGMA) at 2, 6, 10 and 14d following initiation of the culture.

### Amino acid starvation and treatments

Myogenic cells at day 8 of culture were incubated for 72 hours with free amino acid media containing: Earle’s balance salt solution x1 (SIGMA), 20mM HEPES (SIGMA), 9mM NaCO_3_ (SIGMA), Vitamin mix x1 (SIGMA), antibiotics-antimycotic mix x1 (SIGMA) and 4g/L D-glucose (SIGMA), prepared using sterilised MilliQ water filtered at 0.22µm. Starved cells were then growth for 24 h in either free-amino acid media (-AA), media supplemented with recombinant salmon IGF1 (100ng/ml) (GroPep, Australia), medium with complete amino acid (DMEM) or a combination of both. RNA was collected 12 and 24h following treatments using RNeasy mini kit (QIAGEN). For immunofluorescence staining, cells were treated for 48 h and then fixed in 4% (m/v) paraformaldehyde in PBS (SIGMA). Control cells were maintained in DMEM (SIGMA) serum free media.

### Immunofluorescence

Cells were washed in PBS (SIGMA), fixed with 4% (m/v) paraformaldehyde in PBS (SIGMA) for 20 min and made permeable with 0.5% (v/v) X-100 triton (SIGMA)-PBS for 5 min. After PBS washes, cells were blocked for 1h in 5% (v/v) normal goat serum (SIGMA), 1.5% (m/v) Bovine serum albumin (SIGMA), 0.1% (v/v) X-100 triton (SIGMA)-PBS. All antibodies were incubated overnight at 4^o^C in 0.1% (v/v) X-100 triton, 1.5% (m/v) BSA-PBS. Phalloidin-Texas Green antibody (Invitrogen) was diluted 1:1000 (v/v) and anti-desmin (SIGMA) was diluted 1:20 (v/v). For desmin detection cells where incubated with anti-rabbit Alexa Fluor 450 antibody (Invitrogen) for 4h at room temperature. When phalloidin was used, cells were counter-stained for nuclei with DAPI 1:10000 (v/v) (Invitrogen). When desmin was detected cells nuclei were counter-stained with SYTOX-green 1:1000 (v/v). Cells were viewed using a Leica TCS SP2 confocal microscope (Model TCS SP2, Leica Microsystems, Wetzlar, Germany).

### Western Blot Analysis

A total of 20 µg of protein was added to 3 µl of 5X protein loading buffer and 1 µl of 20X reducing agent (Fermentas, Vilnius, Lithuania) and RIPA buffer to 15 µl. Samples were heated for 10 min at 95^o^C and loaded on to a NuPAGE® Novex 4-12% (m/v) poly-acrylamide gel (Invitrogen, Carlsbad, CA, USA). A pre-stained protein ladder ranging from 250 to 10 kDa (BioRad, Hemel, Hetfordshire, UK) was included in all gels to determine the molecular mass of bands. Samples were resolved by electrophoresis for 2h at 100V and room temperature (RT). Proteins were then transferred to a PDVF Immobilon-P Transfer Membrane (Millipore, Billerica, MA, USA) at 25V for 2h at RT. Membranes were washed twice with PBT (0.1% (v/v) Tween 20, SIGMA, in PBS) and blocked for non-specific binding with 5% (m/v) non-fat milk (AppliChem, Darmstadt, Germany) solution in PBT for 1h at RT. After washing in PBT three times for 10 min membranes were incubated at 4^o^C overnight with the following primary antibodies: phospho-Akt (Ser473) (Cell Signalling, #4060, Danvers, MA, USA), Akt (Cell Signalling, #2966), phospho-mTOR (Cell Signalling, #2971) and mTOR (Cell Signal, #2972). Akt and mTOR antibodies were diluted 1:1000 (v/v) and actin in 1:20000 (v/v) in PBT-0.01% (m/v) NaN_3_. Membranes were subsequently incubated with secondary anti-rabbit antibody linked to a horseradish peroxidase (HRP) (SIGMA) diluted 1:40000 (v/v) in 5% (m/v) non-fat milk PBT solution for 1h at RT. After washing in PBT three times for 15 min, membranes were incubated for 1 min with ECL Western Blot detection reagents (GE HealthCare, Amersham, Buckinghamshire, UK). Membranes were exposed to Hyperfilm ECL (GE HealthCare). The resulting films were scanned and band density evaluated with TotalLab Quant software (TotalLab, Newcastle, UK). Phospho-protein levels were normalized with respect to the total protein. A common pooled sample was loaded onto all gels for normalization.

### RNA extraction and cDNA synthesis

Tissue RNA was obtained using TRIsure (Bioline, UK) phenol-chloroform extraction following manufacturer’s recommendations. Cellular RNA was extracted from individual wells using RNeasy plus minikit (Qiagen) following the manufacturer’s recommendations. In all cases RNA concentration, 260/280 and 260/230 ratios were determined using a Nanodrop 1000 Spectophotometer (Thermo, Fisher Scientific, Waltham, MA, USA) and their integrity was confirmed running a 1% (m/v) agarose gel. Only non-degraded RNA samples with 260/280 and 260/230 ratios over 2 were used for cDNA synthesis using a QuantiTec reverse transcription kit (Qiagen). After residual genomic DNA was removed using the DNA wipeout step provided in the kit, 500ng of total RNA were reversed transcribed for 30 minutes at 42^o^C following manufacturer’s recommendations. To detect any contamination of genomic DNA in the samples a reaction with no RT was added to the synthesis reaction (RT-).

### Quantitative Real Time PCR (qPCR)

qPCR was compliant with the Minimum Information for Publication of Quantitative Real-Time PCR experiments MIQE guidelines [32]. Each qPCR mixture contained 6µl of cDNA 1:80 (v/v) diluted, 7.5µl of 2x Brilliant II (Stratagene) and 1.5µl of 500nM primer mix with the following protocol: 1 cycle 5min 95^o^C, 40 cycles 95^o^C 30sec 60^o^C 30sec 72^o^c 30sec and a final cycle of 7min 72^o^C performed in a Stratagene MX3005P real time PCR machine (Stratagene, La Jolla, CA, USA). A gradient was run from 60 to 95^o^C to confirm the presence of single amplification product following dissociation analysis of the PCR products. Products were sequenced to confirm their identity. PCR efficiencies were calculated from dilution series of pooled cDNA samples.

### Primer design

Some of the primers used in the present study have been described previously elsewhere [31,33,34]. Primers were designed using Primer3 [35] with a melting temperature of 60^o^C and, where possible, in region that spanned an exon-exon junction. Primers possible hairpins or non-desirable primer-dimmers were investigated using NetPrimer [36]. Primers used in the present study are summarized in Table S1.

### Data Analysis

GeNorm [37] was used to choose the best reference genes and to analyse the data from each experiment in the study. For the cell culture gene expression analysis ribosomal protein 19 (*rps19*) and Hypoxanthine phosphoribosyl transferase 1 (*hprt1*) were reported as the most stable genes. Elongation factor alpha-1 (*ef1α*) and ribosomal protein L13 (*rspl13*) were identified as the most stable for amino acid deprivation experiment and *rspl13* and *rps29* for the Atlantic salmon fasting re-feeding experiment. GeNorm normalization was performed using the geometric average of the housekeeping genes, and values are shown as arbitrary units.

For data analysis, when data conformed to parametric assumptions an ANOVA using Bonferroni post hoc test was used to detect significant differences. When parametric assumptions were not upheld a Kruskal-Wallis-H test was conducted. Statistical analysis of the data was performed using the SPSS-Statistics (IBM) package.

## Results

### Atlantic salmon possess 6 highly conserved paralogues

**Figure 1:**
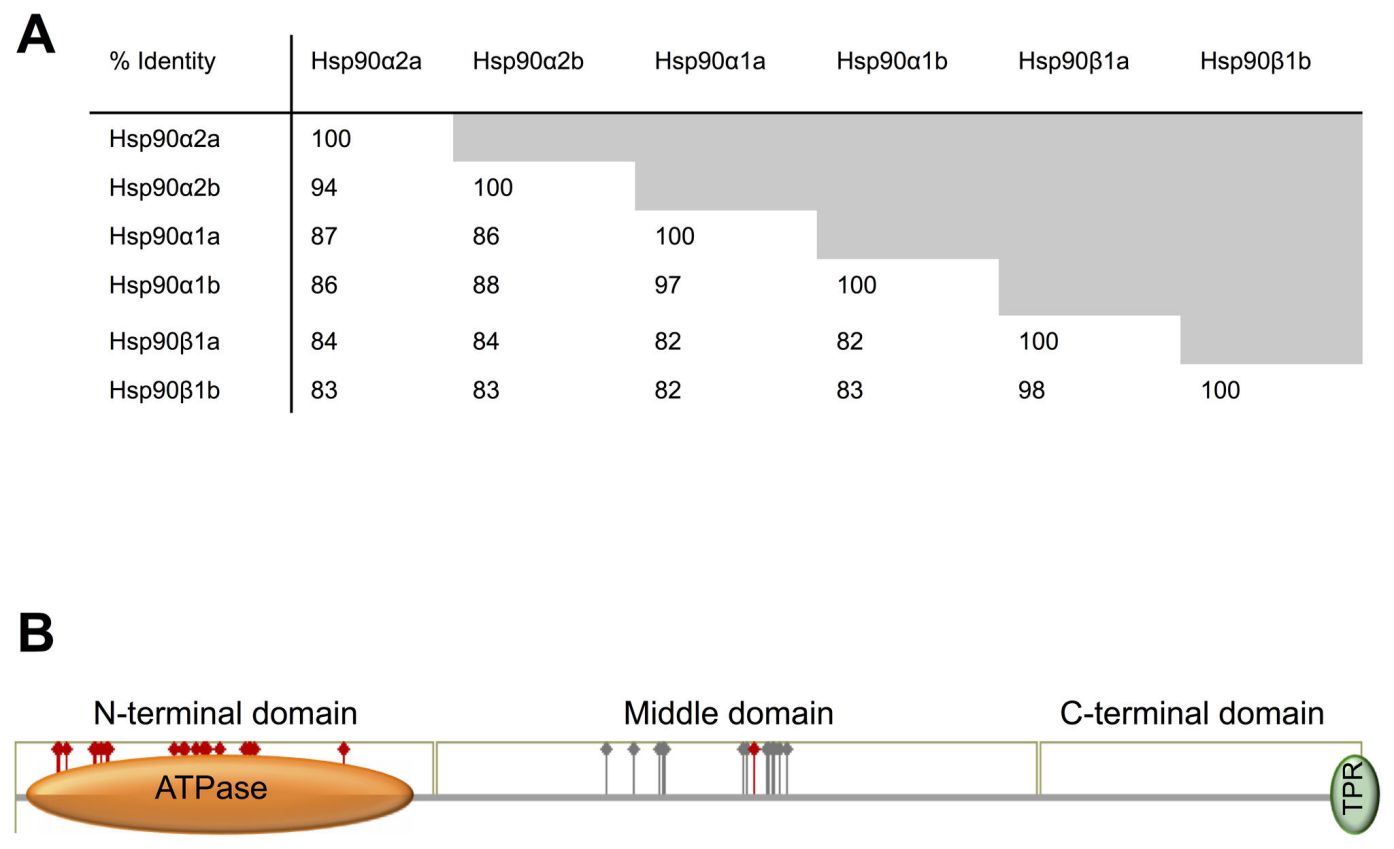
Atlantic salmon Heat shock protein 90 (Hsp90) paralogues. **A**. Identity (%) between amino acid sequences of the Hsp90 paralogues identified for Atlantic salmon **B**. Functional domain organization of the different paralogues. Orange oval represents the extension of the N-terminal ATPase domain. Grey oval identifies the position of the tetratricopeptide motif MEEVD (TRP domain). Red flags highlight amino acids involved in ATP binding conserved in all paralogues. Grey flags highlight residues involved in protein binding conserved in all paralogues. Domains and residues identification are based on previous studies [8,9,47,48].

Previous to this study only two cytosolic Hsp90 were described for Atlantic salmon (*Salmo salar*): Hsp90α (NM_001173702) and Hsp90β (NM_001123532). A total of 6 Hsp90 paralogues, 4 Hsp90α (KC150878, KC150879, KC150880, KC150881) and 2 Hsp90β (KC150882, KC150883) were identified in the Atlantic salmon genome draft [38]. A high degree of amino acid identity was found between groups of paralogues, Hsp90α paralogues shared 87 to 97% identity (Figure 1A) and Hsp90β paralogues shared 98% identity. The percentage identity was reduced to 82-84% when Hsp90ß paralogues were compared to any of the Hsp90α paralogues (Figure 1A, Figure S1).

Characteristic domains of the Hsp90 family were conserved in all Atlantic salmon paralogues (Figure 1B). The N-terminal ATPase domain was localized between amino acid 14 to 300 (Figure 1B, orange domain) and all the residues and motifs previously identified in the literature as key components for the ATP binding and hydrolysis appeared conserved (Figure 1B red flags, Figure S1 red squares). The middle region (between amino acid 300 to 500) was the most variable between paralogues (Figure S1), but residues considered important for binding client proteins were also retained (Figure 1B grey flags, Figure S1 black squares). The MEEVD motif, responsible of the interaction with the tetratricopeptide-repeat domain (TPR)-containing co-chaperones, was also present (Figure 1 green domain, Figure S1 green square).

To confirm the Salmonidae specificity of the paralogues found in Atlantic salmon an extensive rainbow trout ( *Oncorhychus* *mykiss* ) transcriptome was constructed on the bases that both species would share the same number of paralogues. With this objective over 6 millions reads were used to generate a total of 279,336 isotigs (transcriptome details are described in Table S2). Six rainbow trout isotigs with similarity to the Atlantic salmon Hsp90 paralogues were found. Rainbow trout Hsp90 identity and Atlantic salmon relationship was further validated by phylogenetic analysis. Teleost Hsp90α and β orthologues were retrieved from: Atlantic cod (*Gadus morhua*), stickleback ( *Gasterosteus* *aculeatus* ), zebrafish (*Danio rerio*), tilapia ( *Oreochromis* *niloticus* ), medaka (*Oryzias latipes*), fugu ( *Takifugu* *rubripes* ) and green pufferfish (*Tetraodon nigroviridis*) genome sequences.

**Figure 2:**
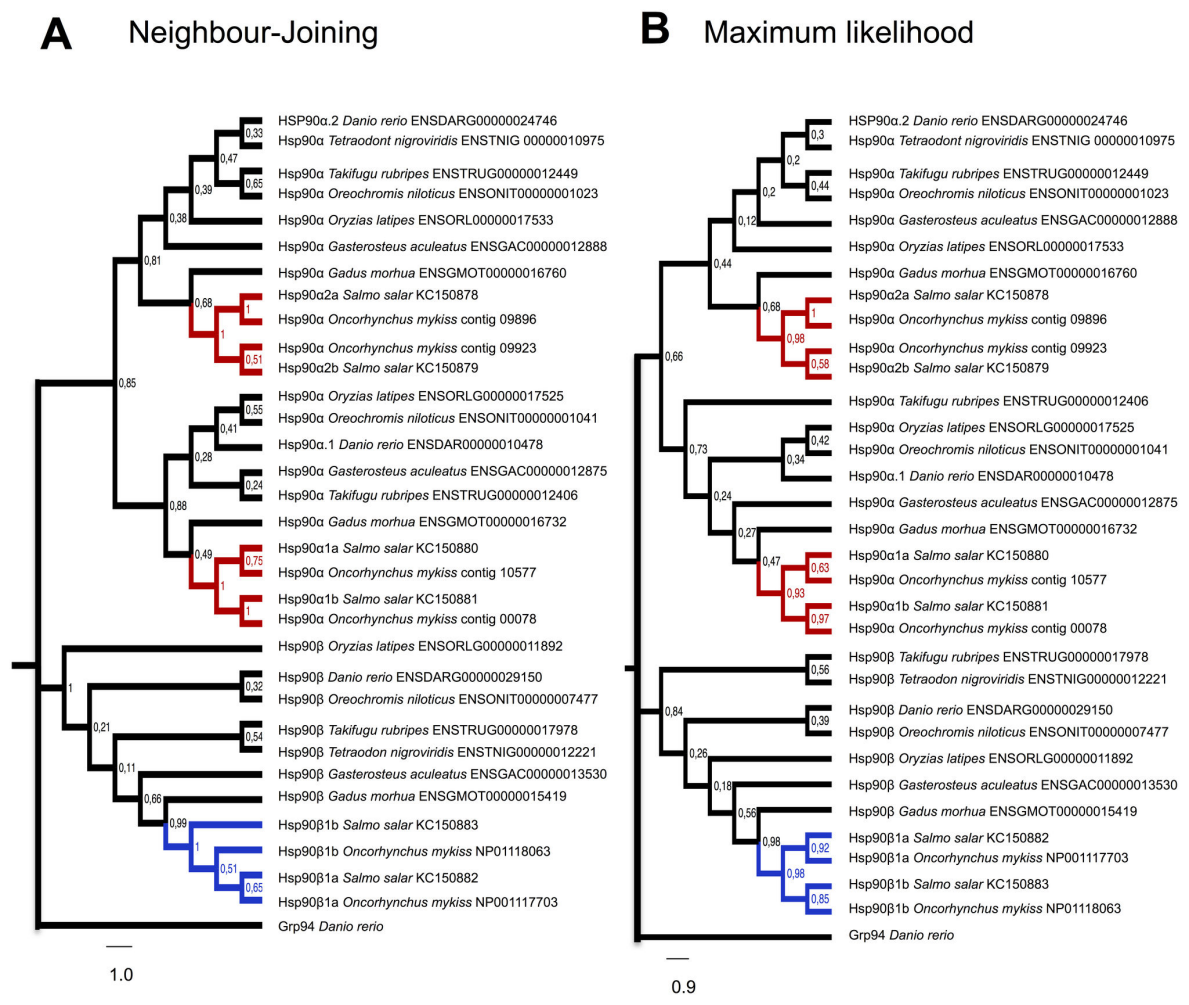
Teleost Hsp90 phylogenetic analysis. Neighbour-Joining (**A**) and Maximum Likelihood (**B**) trees showing phylogenetic relationships between cytosolic Hsp90s from different teleost species. Branches containing Atlantic salmon Hsp90α paralogues are highlighted in red and those with Hsp90β in blue. Phylogenetic trees were constructed from a highly confidence alignment of 37 sequences. Trees were constructed using JJT+G as best fitted evolutionary model. Resulting trees were constructed using MEGA5 with 500 bootstrap repetitions. Bootstrap-posterior values are indicated on the node of each branch.

A total 32 Hsp90 sequences were used producing 674 aligned residues suitable for phylogenetic analysis. NJ and ML trees correctly grouped all sequences into two main clades for Hsp90α and β, confirming the nature of the trout and salmon sequences (Figure 2A and B). Hsp90α sequences were separated in two monophyletic branches with one sequence from each teleost species and two from rainbow trout and Atlantic salmon. Also, the Atlantic salmon Hsp90α paralogues were pairwise branched with individual rainbow trout orthologues (Figure 2, red branches). Based on this analysis, Atlantic salmon Hsp90α paralogues were named according to the zebrafish orthologue branching in the same monophyletic group: Hsp90α1a, Hsp90α1b, Hsp90α2a and Hsp90α2b. Tree topology for Hsp90β orthologues was not well resolved with different topologies between NJ and ML trees (Figure 2, blue branches). Beta paralogues were named as Hsp90ß1a and Hsp90ß1b as have been described for rainbow trout.

**Figure 3:**
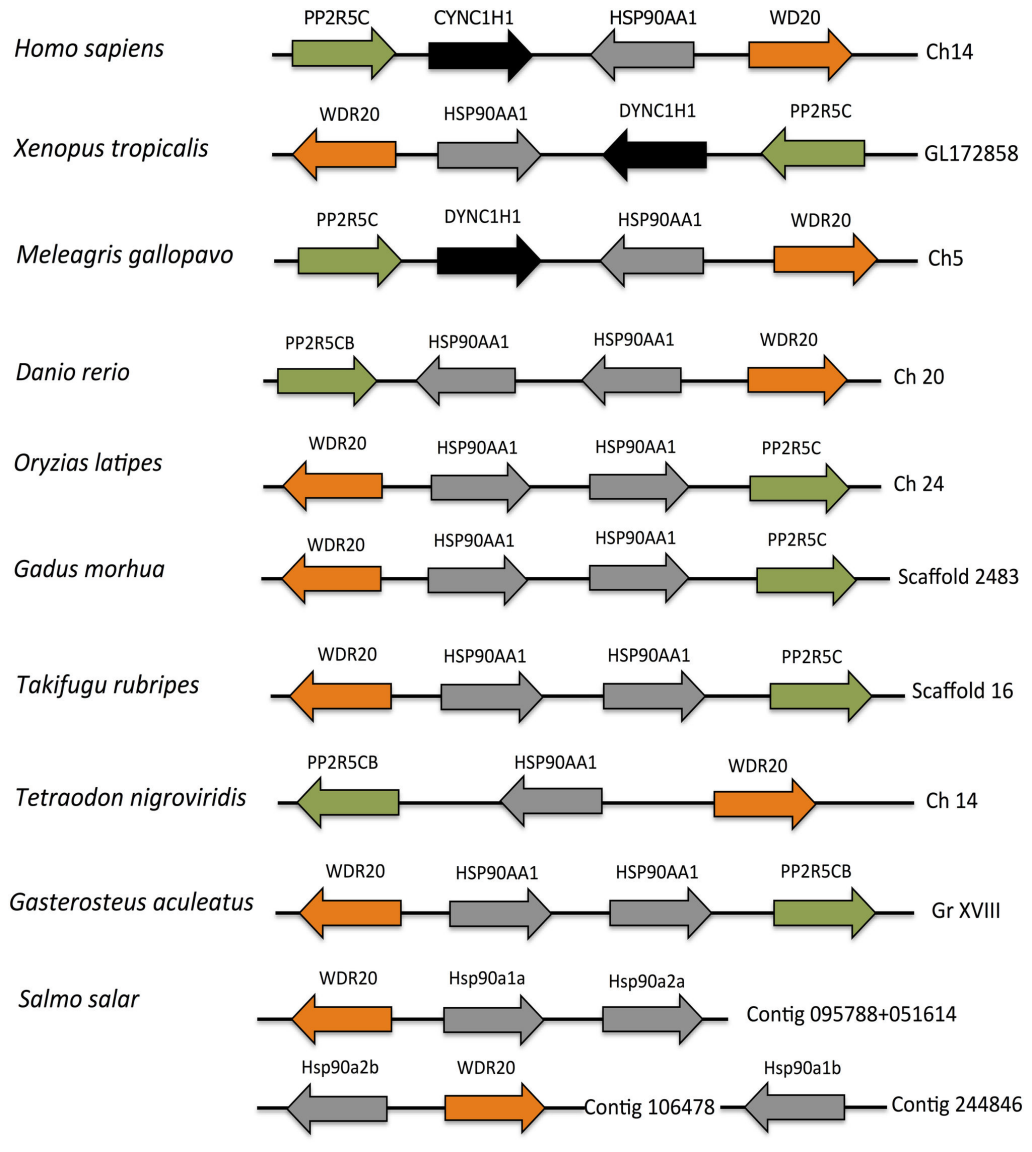
Hsp90 synteny analysis. Hsp90AA1 genetic environment in different vertebrate genomes: *Homo sapiens,* *Meleagris* *gallopavo* *,* *Xenopus* *tropicalis* *, Danio rerio,* *Gasterosteus* *aculeatus* *,* *Takifugu* *rubripes* *, Gadus morhua,* *Tetraodont* *nigroviridis* and *Salmo salar*. Arrow direction refers the orientation of the reading frame. Common genes between genome sections are label with the same colour. Abbreviations are as follow: WD repeat-containing protein 20 (WD20), dynein cytoplasmatic 1 heavy chain 1 (CYNC1H1), serine/threonine-protein phosphatase 2A 56kDa regulatory subunit gamma (PP2R5C), chromosome (Ch), group (Gr).

Synteny analysis showed that, with exception of *Tetraodon* , all basal teleost genomes had two copies of *hsp90α* and one copy of *hsp90β* (Figure 3). Also, in all teleost genomes analysed the Hsp90α paralogues had WD repeat-containing protein 20 (*wdr20*) and serine/threonine-protein phosphatase 2A 56kDa regulatory subunit gamma (*pp2r5c*) as neighbours. Despite the fragmented Atlantic salmon genome it was possible to perform partial synteny analysis by fusing two of the genome-contigs where Hsp90 paralogues were identified. The analysis of these “super-contigs” revealed that *hsp90α1a* and *hsp90α2a* are located next to one another with a copy of *wdr20* next to *hsp90α1a* (Figure 3). We also found a second copy of *wdr20* next to the *hsp90α2b* paralogue (Figure 3). After WGD events, neighbouring genes at the duplicated chromosomes tend to maintain their relative positions or orders during evolutionary time. The finding that two copies of *wdr20* are found next to two *hsp90α* genes suggests the likely WGD origin of the second set of salmon *hsp90α* paralogues.

### Hsp90 paralogues expression is regulated by food intake

**Figure 4:**
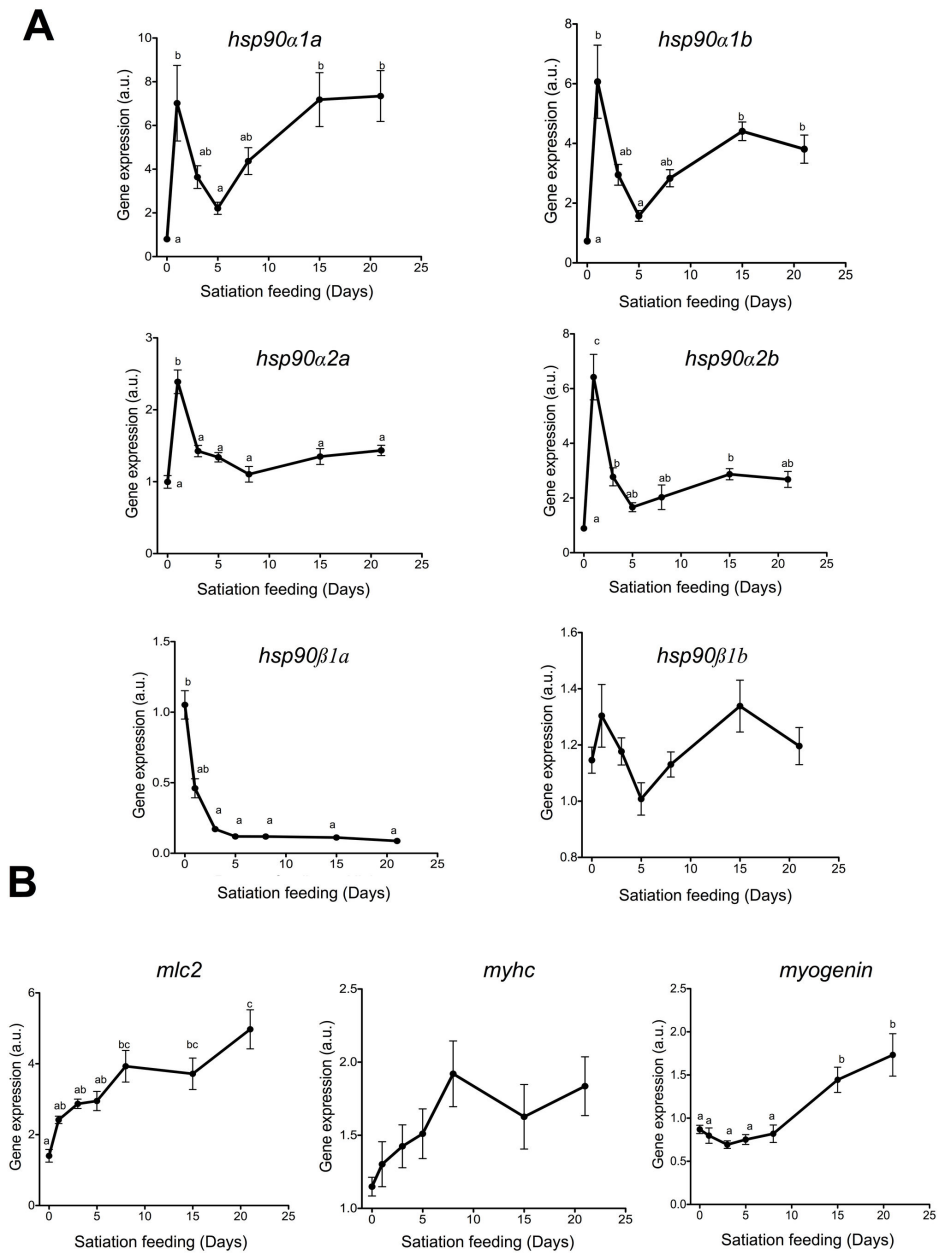
Gene relative expression in Atlantic salmon fast skeletal muscle in response to changes in nutritional status. Effect of fasting for 7d and subsequent refeeding to satiation for 1, 3, 5, 8, 15 and 21 days on gene expression in fast skeletal muscle. Values represent mean ± SE (N=6). Different letters indicate significant differences between means (P<0.05) **A**. *Hsp90α1a, Hsp90α1b, Hsp90α2a, Hsp90α2b, Hsp90ß1a* and *Hsp90ß1b* **B**. Myosin light chain 2 (mlc2), myosin heavy chain (myhc) and *myogenin*.

We investigated the expression of *hsp90* paralogues after manipulating nutritional status in juvenile Atlantic salmon involving a transition from fasting (7d) to satiation feeding (up to 21d). The expression of all *hsp90α* paralogues showed a transient 2 to 8-fold increase in expression peaking 24 h after the initiation of feeding (Figure 4A; P < 0.01). This was followed by a recovery to pre-refeeding levels after 5d. H*sp90α2a* and *2b* expression remained stable over the remainder of the experiment. In contrast, both *hsp90α1* paralogues transcripts increased after 8d and reached a new peak of expression after 15d (4-fold for *hsp90α1b* (P<0.01) and 8-fold for *hsp90α1a* (P<0.01)). This second delayed increase in *hsp90α1a* and *hsp90α1b* expression may reflect the activation of myogenesis and fibre growth induced by refeeding, as previously suggested [19–22]. In support of this idea, and as an indicator of skeletal muscle response, the expression of sarcomeric proteins (myosin light chain 2 (*mlc2*) and myosin heavy chain (*myhc*) and the differentiation factor myogenin increased over a similar timescale (Figure 4B).

### Hsp90 paralogues are differently expressed during maturation of primary myogenic cell cultures

**Figure 5:**
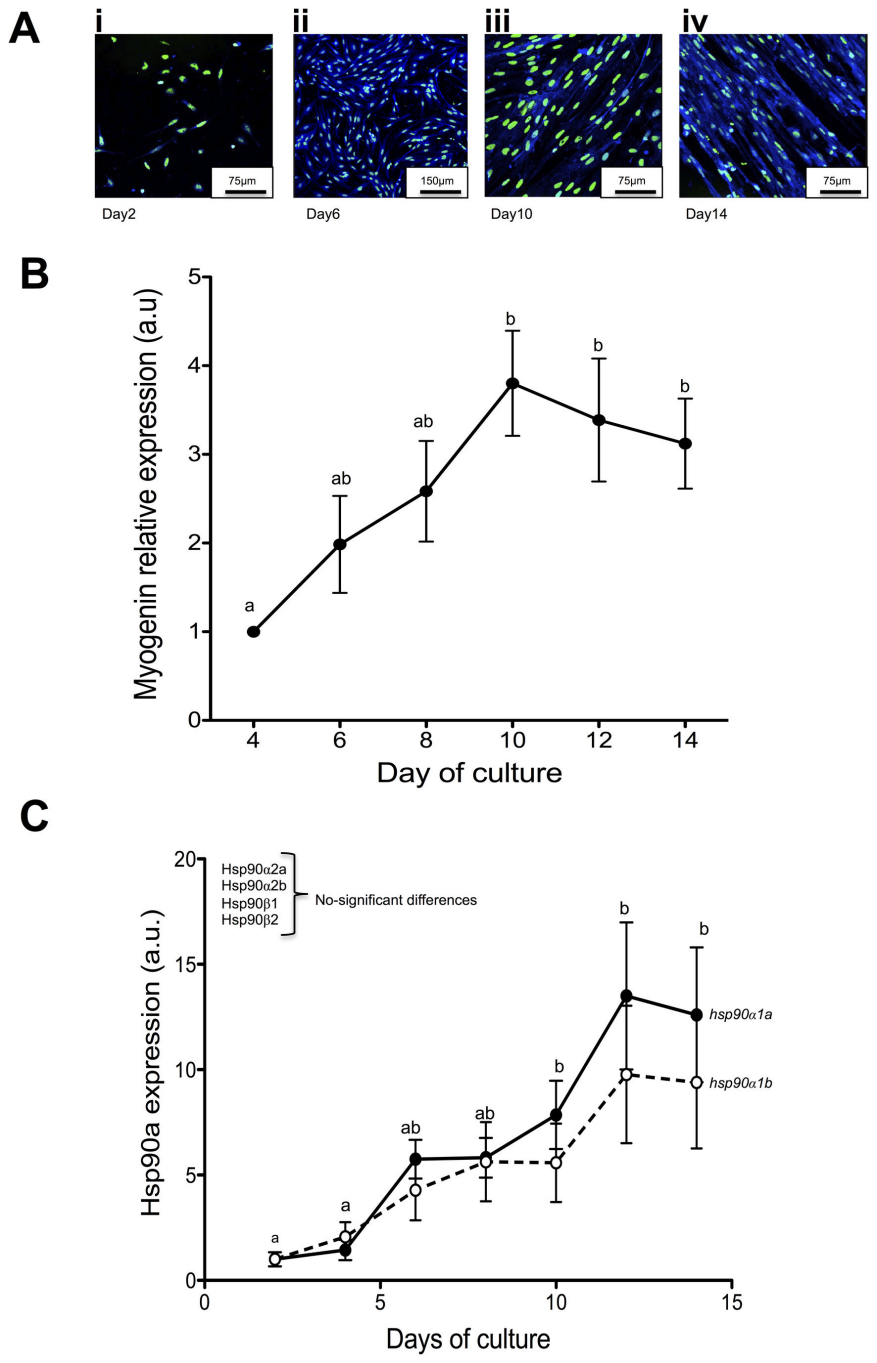
Hsp90 expression in Atlantic salmon myoblast cell culture. **A**. Atlantic salmon fast skeletal muscle myoblasts at days 4 (i), 6 (ii), 10 (iii) and 14 (iv) of culture. Cells nuclei were stained with SYTOX green and myogenic cells were identified by staining for desmin (blue). **B**. *myogenin* expression in myoblast cell culture at days 4, 6, 8, 10, 12 and 14. **C**. *hsp90α1a* and *hsp90α1b* expression in myoblast cell culture at days 2, 4, 6, 10, 12 and 14. To facilitate interpretation of the data, values at day 2 (and 4 for myogenin) were transformed so the mean value = 1. Values represent mean ± SE from 5 different cultures. Different letters indicate significant differences between means (P<0.05).

We then used primary myogenic cell cultures from fast muscle to test the hypothesis that *hsp90α1*, but not *hsp90α2* paralogues, showed increased expression concomitant with myotube formation. Shortly after myoblasts were plated (Figure 5Ai), cells started proliferating until day 5-6 (Figure 5Aii), with the first signs of cell fusion observed between days 8 to 10 (Figure 5Aiii) and fully developed myotubes at day 14 (Figure 5Aiv). Myoblast differentiation was monitored by measuring the expression of the Myogenic Regulatory Factor myogenin which significantly increased it abundance after 10 days of culture (Figure 5B). *Hsp90α1a* and *hsp90α1b* expression increased 10 to- 15 fold (P<0.05) from day 10 concomitant with myotube formation and muscle differentiation (Figure 5C) whereas the expression of other *hsp90* paralogues was unchanged (data not show). It is interesting to note that in both *in vivo* and *in vitro* the Ct values of *hsp90α1a/b* were always much lower than *hsp90α2a/b* (data not show), suggesting higher expression of these paralogues*.*

### Hsp90α paralogues are regulated by amino acid

**Figure 6:**
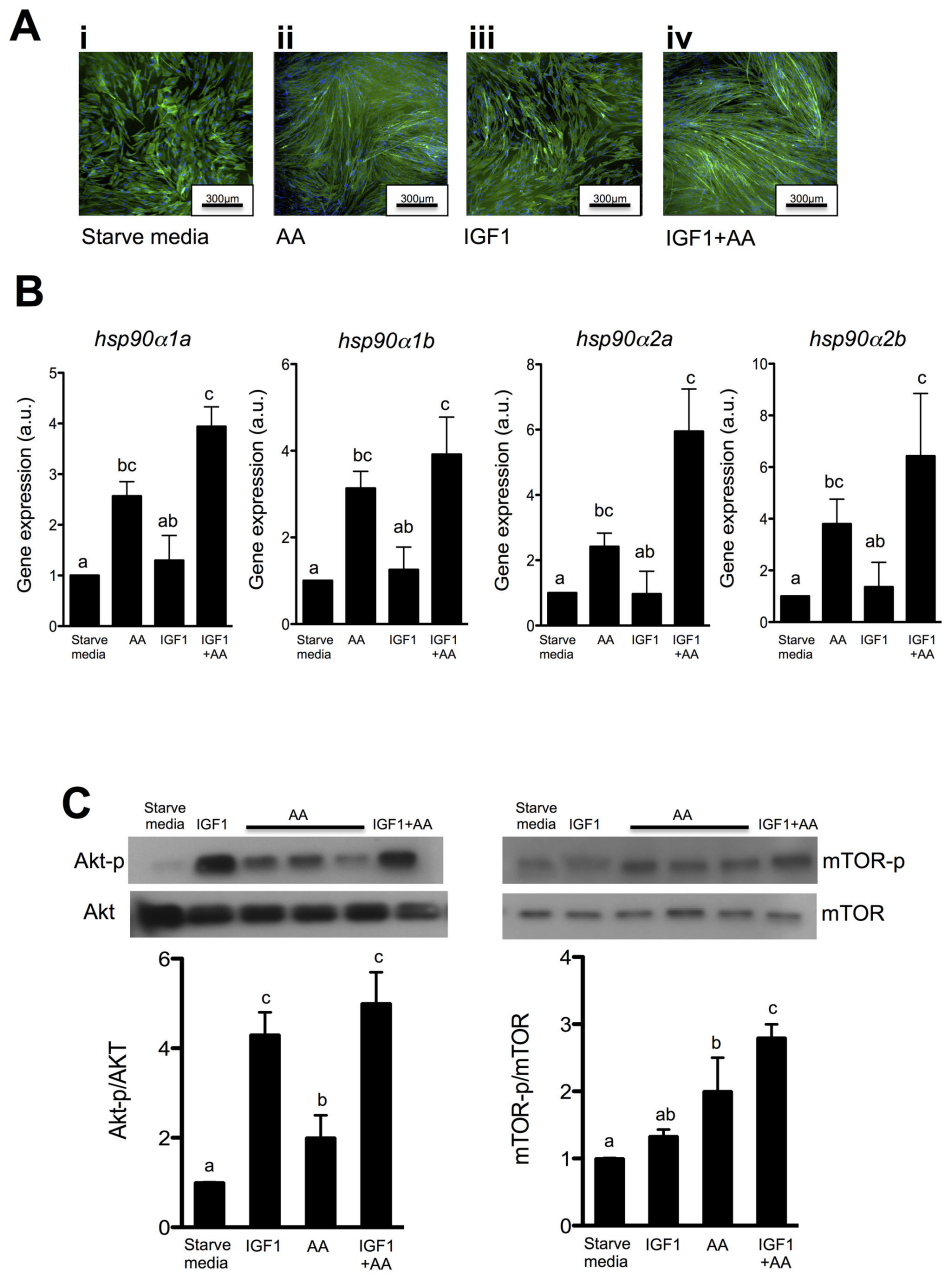
Hsp90 paralogues expression in Atlantic salmon myoblasts incubated with amino acid and IGF1 after 72h of amino acid deprivation. **A**. Actin was stained with green phalloidin and nuclei were counterstained with DAPI (blue) in myoblast incubated with free amino acid medium to simulated starvation (Starve media) (i), with amino acid (AA) (ii), insulin-like growth factor 1 (IGF1) (iii) or amino acid combined with IGF1 (IGF1+AA) (iv). **B**. Expression profiles of *hsp90α1a*, *hsp90α1b*, *hsp90α2a* and *hsp90α2b* in 10 day myogenic cell cultures starved for 72h (Starve media) and then treated with amino acid (AA), insulin-like growth factor 1 (IGF1) or a combination of both (AA+IGF1) for 24h. Values represent mean ± SE from 5 independent cell cultures. For ease of interpretation, values of starved cells (Starve media) were transformed so the mean value = 1. **C**. Akt and mTOR phosphorylation in response to Starve media, AA, IGF1 and AA+IGF1 treatments. Relative units were obtained by normalizing the phosphorylated-protein to the total protein. Values represent mean ± SE from 5 different cultures. Different letters indicate significant differences between means (P<0.05).

To further explore the transcriptional regulation of *hsp90* paralogues by nutrition their expression was analysed in 8 d cultures following treatment with amino acids and IGF1. Cultures were transferred to serum/amino acid free medium (“starved media”) for 72h and then incubated with medium containing either amino acid (AA), IGF1 or a combination of both (IGF1+AA). Akt and mTOR phosphorylation was also measured to provide evidence for the activation of the PI3K and mTor pathways respectively (Figure 6C). Myoblasts incubated with amino acid/serum-free medium stopped their progression through myogenesis and failed to fuse to form myotubes (Figure 6Ai). Myotubes formed again after 48h in the presence of amino acid alone or in combination with IGF1 (Figure 6Aii and Aiv). IGF1 alone failed to recover myotube formation (Figure 6Aiii). Phosphorylation of Akt increased 4-5 fold in the presence of IGF1 indicating activation of PI3K signalling. In contrast, phosphorylation of mTor was unchanged by IGF1 treatment, but increased 2-3 fold when amino acid were added alone or in combination with IGF1, consistent with the activation of mTor signalling (Figure 6C).

Significant changes in gene expression were observed 24h after treatments. When amino acid were added to the medium all *hsp90α* paralogues showed an almost 3-fold increase in expression (P<0.05). *Hsp90α* expression was unchanged in myoblasts treated with IGF1 alone. However, when amino acids were combined with IGF1, all 4 *hsp90α* paralogues significantly increased their expression (Figure 6B; P < 0.05 for *hsp90α2a/b*; P<0.01 for *hsp90α1a/b*) to levels greater than observed with amino acids alone. *Hsp90β* expression was not affected by any of the treatments (data not show).

Our working hypothesis is that amino acid influx to the muscle stimulates the mTor pathway leading to increased peptide translation and concomitant activation of the protein folding response pathway. To investigate this further the expression of other chaperones, co-chaperones and protein editing genes including *hsp70*, *hsp30*, *tbcb*, *sgta*, *fkbp4*, *pdia4* and *pdia6* were analysed under the same *in vitro* conditions (Table 1). *Hsp30*, *sgta*, *tbcb* and *fkbp4* increased their expression 2 to 3-fold (P<0.05) in response to amino acid combined with IGF1 after 24h (Table 1). Expression of the disulfide-isomerases *pdia4* and *pdia6* showed a significant response after 12h to amino acid alone (2-4 fold; P<0.05) or when were combined with IGF1 (3-5 fold; P<0.05) (Table 1). In contrast, there were no changes in *rab9a* expression and, although *hsp70* levels were significantly altered, expression did not follow a consistent pattern (Table 1).

**Table 1:** Expression profiles of folding and protein-editing genes in Atlantic salmon myoblasts incubated with amino acid and insulin-like growth factor 1 (IGF1) after 72h of amino acid deprivation.

**Time**	**12h**				**24h**			
**Condition**	**Starvation media**	**AA**	**IGF1**	**AA+IGF1**	**Starvation media**	**AA**	**IGF1**	**AA+IGF1**
**Gene ID**								
*sgta*	*nd*	*nd*	*nd*	*nd*	1.0 ± 0.0	1.98 ± 0.3	0.84 ± 0.1	3.36 ± 0.3*
*fkbp4*	*nd*	*nd*	*nd*	*nd*	1.0 ± 0.0	1.89 ±0.1	0.79 ± 2.1	2.17 ± 0.1*
*tbcb*	*nd*	*nd*	*nd*	*nd*	1.0 ± 0.0	1.79 ± 0.2	0.97 ± 0.1	2.66 ± 0.2*
*hsp30*	*nd*	*nd*	*nd*	*nd*	1.0 ± 0.0	1.54 ± 0.4	0.70 ± 0.2	2.89 ± 0.1*
*pdia4*	1.0 ± 0.0	2.68 ± 0.9	1.06 ± 0.3	3.37 ± 0.9*	*nd*	*nd*	*nd*	*nd*
*pdia6*	1.0 ± 0.0	4.43 ± 1.8*	1.55 ± 0.9	5.67 ± 1.5*	*nd*	*nd*	*nd*	*nd*
*hsp70*	1.0 ± 0.0	5.53 ± 1.7*	2.25 ± 0.7	4.03 ± 1.0*	1.0 ± 0.0	2.65 ± 1.4	5.3 ± 1.1*	5.02 ± 1.3*

Values are expressed as mean ± SE from 5 independent Atlantic salmon myoblast cell cultures. Significant differences between treatments for each gene with respect to the cells in “starved media” are indicated with an asterisk (*) (P<0.05).

nd: no statistical difference

Starved media: cells incubated with amino acid free medium

AA: cells incubated with medium containing amino acid

IGF1: cells incubated with medium containing 100ng/ml recombinant Atlantic salmon IGF1

AA+IGF1: cells incubated with a combination of amino acid and IGF1

## Discussion

Our synteny analysis of teleost genomes has shown that the majority of studied species apart from *Tetraodon* *nigriviridis* have two copies of *hsp90α* and one copy of *hsp90ß* (Figure 3). *Hsp90α* paralogues were always found next to each other on the same chromosome/contig and flanked by *wdr20* and *pp2r5c* genes (Figure 3). These results indicate that Hsp90α paralogues originated by tandem duplication at the base of the teleost linage (Figure 3). Salmonid genomes contain four paralogues of Hsp90α and two Hsp90ßs which were named *hsp90α1a, hsp90α1b, hsp90α2a, hsp90α2b, hsp90ß1a* and *hsp90ß1b* based on a phylogenetic analysis (Figure 2). Despite fragmented synteny an examination of the available contigs allowed us to make two conclusions; 1: that *hsp90α1a* and *hsp90α2a* are found next to one another as observed in other teleost genomes, indicating they originated by tandem duplication and 2: the presence of two copies of *wd20* adjacent to *hsp90α1a* and *hsp90α2b* (Figure 3) suggests that the second pair of Hsp90α salmon paralogues probably originated during the salmonid-specific WGD [24] since neighbouring genes on the duplicated chromosomes tend to maintain their relative positions or orders over evolutionary time [39]. It has previously been suggested that proteins which form homodimers, such as Hsp90, are more likely to be retained after WGD [40].

Research on Hsp90s in teleosts has mainly focused on their roles in temperature stress [16–18] and embryogenesis [14,41,42] including skeletal muscle formation [15]. Studies in Atlantic salmon [20], zebrafish [21] and gilthead sea bream [22], have reported that *hsp90α* expression is regulated by feeding in agreement with our findings (Figure 4). In the present study *in vitro* experiments have provided direct evidence for the stimulation of *hsp90α1a* and *hsp90α1b* expression by amino acids (Figure 6). Furthermore the growth factor IGF1, which stimulates protein synthesis via the PI3K pathway in muscle [43], was not able to stimulate *hsp90α* expression unless amino acids were also present (Figure 6B). Our data suggests that *hsp90α* regulation by amino acids acts in synergy with the PI3K/Akt pathway, but is independent of Akt phosphorylation (Figure 6). Amino acids (alone or combined with IGF1) were able to increase mTor phosphorylation (Figure 6) in agreement with the general idea that mTor acts as an intracellular nutrient sensor [44]. mTor has been reported to regulate *hsp90* expression in response to stress through the phosphorylation of Heat shock factor 1 (HSF1) [45]. Other in vitro studies with salmon myoblasts similarly reported that, in absence of amino acids, IGF1 was unable to increase the expression of several genes related with muscle growth including *pcna*, *pax7*, *myod1b*, *myod1c*, *igf1* itself, *igf2*, *igfbp4*, *igfbp5*.*1* and *igfbp5.2* [31,33]. In the present study other chaperones (*hsp30*, *hsp70*, *tcbc*), co-chaperones (*sgta*, *fkbp4*) and protein-disulphide isomerases (*pdia4*, *pdia6*) associated with Hsp90 and protein editing also showed increased expression with amino acid treatment, indicating a general activation of the protein folding response (Table 1).

Our results suggest that all four *hsp90α* share regulatory elements that are activated in response to changes in intracellular amino acid concentration (Figures 4 and 6), whereas only *hsp90α1a* and *hsp90α1b* were associated with myotube formation and muscle differentiation (Figures 4 and 5). *Hsp90α1* paralogues are required for the correct formation of the skeletal muscle in zebrafish embryos [15]. It is therefore likely that salmon *hsp90α1* paralogues retains analogous myogenesis-related functions to those describes for the zebrafish *hsp90α1* orthologue.

It has been widely accepted that *Hsp90α* is the facultative form of cytosolic Hsp90 and that *hsp90ß* is constitutive expressed [46]. We found that *hsp90ß1a* and *ß1b* did not change their expression during maturation of primary myogenic cultures, whereas *hsp90ß1a* was significantly downregulated between the fasting and feeding states *in vivo* (Figure 4A). In gilthead sea bream *hsp90ß* was also elevated during fasting and returned to lower values after re-feeding [22]. Since *hsp90ß1a* was not affected when cells were incubated in a free amino acid/hormone medium it seems that is not the absence of nutrients or serum that regulates *hsp90ß1a* expression, but rather some as yet uncharacterised signal(s) from fasting, such as glucagon, ghrelin or other hormones.

## Conclusions

In the present study we found that Atlantic salmon possess an expanded set of Hsp90 paralogues that probably originated during the salmonidae-specific WGD. We have demonstrated that the *hsp90α* paralogues together with other chaperones and co-chaperones increase their expression in response to amino stimulation during feeding, likely involving enhanced mTor signalling. The present study suggests specific roles for *Hsp90α1a/b* in muscle differentiation based on *in vivo* and *in vitro* expression patterns. We also show that H*sp90ß1b* expression is regulated by signals other than amino acids whereas Hsp90ß1a is constitutively expressed as is the single Hsp90ß in mammals and zebrafish.

## Supporting Information

Figure S1 **Hsp90 amino acid sequence alignments.** The ATPase domain is indicated with an underscored red line, middle-domain with a low blue line and C-terminal domain with an underscored green line. Motifs and amino acid involved in ATP binding and hydrolysis are highlighted in red. Residues implicated in protein binding are highlighted in black. The MEEVD motif is highlighted in green.
(TIFF)

Table S1 **Quantitative PCR primer sequences, PCR efficiencies and correlation coefficients of standard curves for genes.** E, PCR efficiency; Tm, melting temperature; F, forward; R, reverse. Genes are as follow: heat shock protein 90 kDa (*hsp90*), Heat shock protein 70kDan (*hsp70*), heat shock protein 30kDa (*hsp30*), small glutamine-rich tetratricopeptide repeat-containing protein alpha (*sgta*), protein disulfide-isomerase A (pdia), myosin heavy chain (*Myhc*), myosin light chain 2 (*mlc2*), beta actin (β-actin), RNA polymerase 2 (*rnapol II*), elongation factor alpha (*ef1a*), hypoxanthine-guanine phosphoribosyltransferase 1 (*hprt1*), 40S ribosomal protein S19 (*rps19*), 60S ribosomal protein L13 (*rpl13*), tubulin folding cofactor b (tbcb) and fk506 binding protein 4 (*fkbp4*).
(DOCX)

Table S2 **Rainbow trout *de novo* transcriptome metrics.** bp: base pair.
Singletons: reads not contained in the final assembly.
Isotig: contigs consistently connected by a set of reads.
N50: The value was computed by sorting all contigs from largest to smallest and by determining the minimum set of contigs whose sizes total 50% of the entire transcriptome.
(DOCX)
