# Supplementary figures and images for: Expression of Heat Shock Protein (Hsp90) Paralogues Is Regulated by Amino Acids in Skeletal Muscle of Atlantic Salmon
Source: PLoS One. 2013 Sep 6;8(9):e74295. doi: 10.1371/journal.pone.0074295 (PMC3765391; doi:10.1371/journal.pone.0074295)

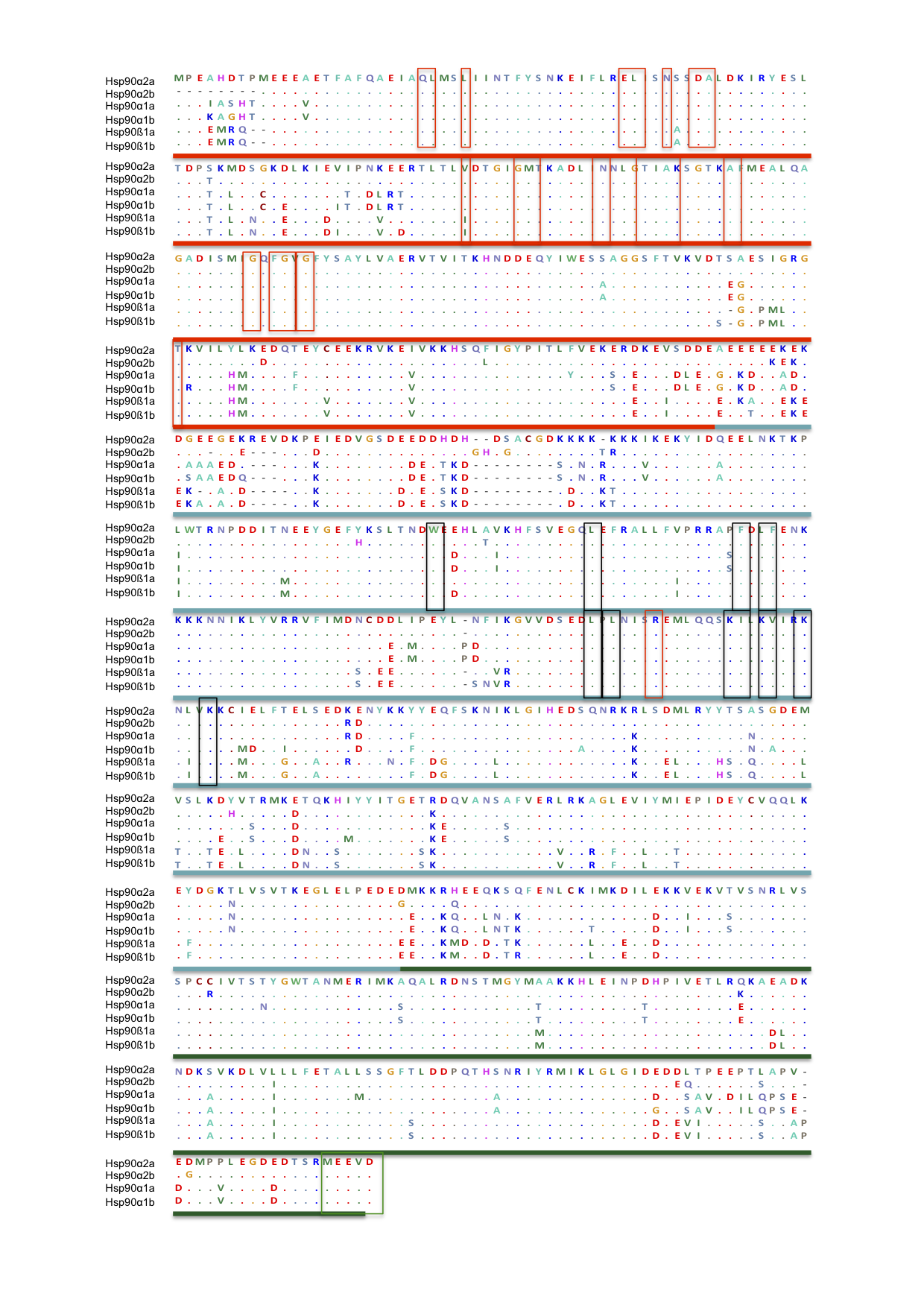

Supplement: Figure S1 — Hsp90 amino acid sequence alignments. The ATPase domain is indicated with an underscored red line, middle-domain with a low blue line and C-terminal domain with an underscored green line. Motifs and amino acid involved in ATP binding and hydrolysis are highlighted in red. Residues implicated in protein binding are highlighted in black. The MEEVD motif is highlighted in green. (TIFF) [file pone.0074295.s001.tiff]
