# Supplementary material for: Expression of Heat Shock Protein (Hsp90) Paralogues Is Regulated by Amino Acids in Skeletal Muscle of Atlantic Salmon
Source: PLoS One. 2013 Sep 6;8(9):e74295. doi: 10.1371/journal.pone.0074295 (PMC3765391; doi:10.1371/journal.pone.0074295)
Supplement: Table S1 — Quantitative PCR primer sequences, PCR efficiencies and correlation coefficients of standard curves for genes. E, PCR efficiency; Tm, melting temperature; F, forward; R, reverse. Genes are as follow: heat shock protein 90 kDa (hsp90), Heat shock protein 70kDan (hsp70), heat shock protein 30kDa (hsp30), small glutamine-rich tetratricopeptide repeat-containing protein alpha (sgta), protein disulfide-isomerase A (pdia), myosin heavy chain (Myhc), myosin light chain 2 (mlc2), beta actin (β-actin), RNA polymerase 2 (rnapol II), elongation factor alpha (ef1a), hypoxanthine-guanine phosphoribosyltransferase 1 (hprt1), 40S ribosomal protein S19 (rps19), 60S ribosomal protein L13 (rpl13), tubulin folding cofactor b (tbcb) and fk506 binding protein 4 (fkbp4). (DOCX) [file pone.0074295.s002.docx]

Table S1. Quantitative PCR primer sequences, PCR efficiencies and correlation coefficients of standard curves for genes.

| Gene Name | Primers (5’-3’) | E (%) | Size (bp) | Tm (Cº) | Accession number |
| --- | --- | --- | --- | --- | --- |
|  |  |  |  |  |  |
| *hsp90α2a* | F:AAGGAGAAGGATGGGGAAGAAGGA  R:TTCTTCTTCTTCTTCTTGTCACCG | 95 | 250 | 82.3 | KC150878 |
|  |  |  |  |  |  |
| *hsp90α2b* | F:GAGAAGAAGGATGGGGAAGGAGAG  R:CTTGTCCCCACATGCGCCATCG | 96 | 108 | 83.8 | KC150879 |
|  |  |  |  |  |  |
| *hsp90α1a* | F:AAAAAAACAGGAGGAGCTGAATT  R:ATGTTGGCTGTCCACCCGTAGTTG | 99 | 156 | 82.8 | KC150880 |
|  |  |  |  |  |  |
| *hsp90α1b* | F:GGATGAGGATGAGAAGAAGAAGCAA  R:ATGTTGGCCGTCCAGCCGTATGTA | 97 | 171 | 82 | KC150881 |
|  |  |  |  |  |  |
| *hsp90β1* | F:TGGATGAGGACAAGACAAAGTTCG  R:GCTGAAGCCAGAGGAGAGGAGA | 100 | 350 | 86 | KC150882 |
|  |  |  |  |  |  |
| *hsp90β2* | F:AGGAGGACAAGACGAGGTTTGA  R:GCTGAAGCCCGAAGAGAGCAATG | 102 | 345 | 86.3 | KC150883 |
| *hsp30* | F: CCGTTCAGGCAGATCAAACT  R: GAGGAGCTGTCTGTCAAGCA | 94 | 135 | 82 | NP_001134440 |
|  |  |  |  |  |  |
| *myhc* | F: GCACGCCACTGAAAAC  R: CCTCAAGGTCGTCCACT | 95 | 209 | 84 | DN164736 |
| *mlc2* | F: TCAACTTCACCGTCTTCCTCAC  R: GCCCACAGGTTCTTCATCTCC | 98 | 194 | 83 | NM_001123716 |
| *myogenin* | F: GTGGAGATCCTGAGGAGTGC  R: CTCACTCGACGACGAGACC | 99 | 146 | 85 | DQ294029 |
| *hsp70* | F: CCTGGTGAAGATGAGGGAGA  R: GTTCCCTGGACATGCCTTG | 103 | 106 | 81 | AC134374 |
|  |  |  |  |  |  |
| *sgta* | F:AGCGTCTTGCCTTCTCCAT  R:GCAGAGGCAAAAATCTCTGG | 92 | 181 | 82.8 | BT045931.1 |
|  |  |  |  |  |  |
| *pdia4* | F:ATCGCTGACGAGGAGGACTA  R:TTCCCTCAGCACCTCAGAGT | 93 | 147 | 84.5 | BT072419.1 |
|  |  |  |  |  |  |
| *pdia6* | F:ACTGAGGCTGGTGCTCAGAT  R:GATCCCTGAGGAACTCGTGA | 96 | 154 | 87 | BT046102.1 |
|  |  |  |  |  |  |
| *fkbp4* | F:ATGAAGGAGCTGCCGTAGAA  R:GAGGGACTCTTCTCCCTGCT | 96 | 161 | 85 | NM_001140424.1 |
|  |  |  |  |  |  |
| *tbcb* | F:TTGTAGGGCTACCCACCAAG  R:CACACTGCCATCATGTTTCC | 100 | 118 | 81 | NM_001140492.1 |
|  |  |  |  |  |  |
| *rpl13* | F: CGCTCCAAGCTCATCCTCTTCCC  R: CCATCTTGAGTTCCTCCTCAGTGC | 96 | 79 | 84 | BT043698 |
|  |  |  |  |  |  |
| *ef1a* | F:GAATCGGCTATGCCTGGTGAC  R:GGATGATGACCTGAGCGGTG | 101 | 141 | 86 | BG933853 |
|  |  |  |  |  |  |
| *rps19* | F: GGGTCATCAGCAGCTCTATTGG,  R: AGTCCAGCTTAACAAAGCCGATG | 98 | 167 | 85 | NM_001139600 |
|  |  |  |  |  |  |
| *hprt1* | F:CCGCCTCAAGAGCTACTGTAAT  R:GTCTGGAACCTCAAACCCTATG | 98 | 255 | 82 | EG866745 |
|  |  |  |  |  |  |
| *ß-actin* | F: TGACCCAGATCATGTTTGAGACC  R: TGACCCAGATCATGTTTGAGACC | 97 | 146 | 84.8 | G933897 |
|  |  |  |  |  |  |
| *rnapol II* | F: CCAATACATGACCAAATATGAAAGG  R: ATGATGATGGGGATCTTCCTGC | 107 | 157 | 84.5 | BG936649 |

E, PCR efficiency; Tm, melting temperature; F, forward; R, reverse. Genes are as follow: heat shock protein 90 kDa (*hsp90*), Heat shock protein 70kDan (*hsp70*), heat shock protein 30kDa (*hsp30*), small glutamine-rich tetratricopeptide repeat-containing protein alpha (*sgta*), protein disulfide-isomerase A (*pdia*), myosin heavy chain (*Myhc*), myosin light chain 2 (*mlc2*), beta actin (ß-actin), RNA polymerase 2 *(rnapol II*), elongation factor alpha (*ef1a*), hypoxanthine-guanine phosphoribosyltransferase 1 (*hprt1*), 40S ribosomal protein S19 (*rps19*), 60S ribosomal protein L13 (*rpl13*), tubulin folding cofactor b (tbcb) and fk506 binding protein 4 (*fkbp4*) .
