# Supplementary material for: Expression of Heat Shock Protein (Hsp90) Paralogues Is Regulated by Amino Acids in Skeletal Muscle of Atlantic Salmon
Source: PLoS One. 2013 Sep 6;8(9):e74295. doi: 10.1371/journal.pone.0074295 (PMC3765391; doi:10.1371/journal.pone.0074295)
Supplement: Table S2 — Rainbow trout de novo transcriptome metrics. bp: base pair. Singletons: reads not contained in the final assembly. Isotig: contigs consistently connected by a set of reads. N50: The value was computed by sorting all contigs from largest to smallest and by determining the minimum set of contigs whose sizes total 50% of the entire transcriptome. (DOCX) [file pone.0074295.s003.docx]

Table S2. Rainbow trout transcriptome metrics

| *Transcriptome parameters* |  |
| --- | --- |
| Reads used | 6,154,973 |
| Number of bases (bp) | 3,085,524,059 |
| Reads aligned | 4,716,451 |
| Number of bases aligned (bp) | 2,016,261,631 |
| Number of isotigs | 279,336 |
| Average isotig size (bp) | 1,172 |
| N50 isotig size (bp) | 1,891 |
| Singletons | 178,846 |

bp: base pair

Singletons: reads not contained in the final assembly.

Isotig: contigs consistently connected by a set of reads.

N50: The value was computed by sorting all contigs from largest to smallest and by determining the minimum set of contigs whose sizes total 50% of the entire transcriptome.
